# Cross-frequency interaction of the eye-movement related LFP signals in V1 of freely viewing monkeys

**DOI:** 10.3389/fnsys.2013.00001

**Published:** 2013-02-14

**Authors:** Junji Ito, Pedro Maldonado, Sonja Grün

**Affiliations:** ^1^Institute of Neuroscience and Medicine (INM-6), Computational and Systems Neuroscience, Forschungszentrum JülichJülich, Germany; ^2^BNI, CENEM and Programa de Fisiología y Biofísica, ICBM, Facultad de Medicina, Universidad de ChileSantiago, Chile; ^3^Theoretical Systems Neurobiology, RWTH Aachen UniversityAachen, Germany; ^4^RIKEN Brain Science InstituteWako-Shi, Japan

**Keywords:** local field potential, oscillation, saccade, natural vision, cross-frequency coupling

## Abstract

Recent studies have emphasized the functional role of neuronal activity underlying oscillatory local field potential (LFP) signals during visual processing in natural conditions. While functionally relevant components in multiple frequency bands have been reported, little is known about whether and how these components interact with each other across the dominant frequency bands. We examined this phenomenon in LFP signals obtained from the primary visual cortex of monkeys performing voluntary saccadic eye movements (EMs) on still images of natural-scenes. We identified saccade-related changes in respect to power and phase in four dominant frequency bands: delta-theta (2–4 Hz), alpha-beta (10–13 Hz), low-gamma (20–40 Hz), and high-gamma (>100 Hz). The phase of the delta-theta band component is found to be entrained to the rhythm of the repetitive saccades, while an increment in the power of the alpha-beta and low-gamma bands were locked to the onset of saccades. The degree of the power modulation in these frequency bands is positively correlated with the degree of the phase-locking of the delta-theta oscillations to EMs. These results suggest the presence of cross-frequency interactions in the form of phase-amplitude coupling (PAC) between slow (delta-theta) and faster (alpha-beta and low gamma) oscillations. As shown previously, spikes evoked by visual fixations during free viewing are phase-locked to the fast oscillations. Thus, signals of different types and at different temporal scales are nested to each other during natural viewing. Such cross-frequency interaction may provide a general mechanism to coordinate sensory processing on a fast time scale and motor behavior on a slower time scale during active sensing.

## Introduction

Living organisms have the ability to actively explore their surroundings using their sensory organs. Such behavior is called active sensing and it includes for instance, sniffing for odor sensation (Uchida and Mainen, 2003), whisking for touch sensation (Kleinfeld et al., 2006), and eye movements (EMs) for visual sensation (Land, 1999). All these sensing behaviors are performed as rhythmic repetitions of short, discrete sampling actions. Intriguingly, the sampling frequencies in active sensing are similar across different sensory modalities; the frequencies typically lie within the delta-theta frequency band (2–10 Hz) (Schroeder et al., 2010; Cao et al., 2012). This may indicate that this frequency band reflects an optimal time scale for the coordination of the brain activities in the sensory and the motor systems required for active sensing (Kleinfeld et al., 2006; Uchida and Kepecs, 2006; Schroeder et al., 2010).

In natural vision, primates achieve discrete sampling of the visual environment by the combination of visual fixations (with typical durations of 200–400 ms) and ballistic EMs, called saccades, that direct the gaze from one fixation location to the next. While the neural mechanisms underlying the execution of saccades and the neural activities in the visual cortices around the time of saccades have been extensively studied, those related to active visual sensing, i.e., visual exploration with voluntary, successive saccades, have remained largely unknown. Recently, Ito et al. (2011) showed in monkeys freely viewing natural-scene images that the local field potential (LFP) in V1 expresses oscillatory modulations in the beta frequency band (10–25 Hz) that are locked to the onset of saccades. They also found that the onset of the visually evoked spiking activity is phase-locked to these LFP modulations, which was identified as the mechanism for the occurrence of excess spike synchrony found among V1 neurons (Maldonado et al., 2008). These results suggest a functional relevant role of the beta band LFP oscillations in the coordination of EMs and visual sensory processing during natural viewing behaviors.

Other studies have also demonstrated instrumental roles of oscillatory brain signals in natural vision. Belitski et al. (2008) and Mazzoni et al. (2011) showed that during passive viewing of natural movies while maintaining prolonged fixation on a central spot (i.e., Without EMs), the delta-theta frequency band (1–8 Hz) component of the LFP in V1 was modulated coherently with the temporal changes in the contrast level of the movies, on the same time scale as the LFP oscillations. Thus, LFP signals carry information about the slow changes in the visual stimulus, which is not contained in the spiking activities in the same area. Rajkai et al. (2008) reported modulations in current-source density signals in the delta-theta frequency band related to spontaneous EMs in the dark, and Bosman et al. (2009) reported micro-saccade related LFP oscillations in the same frequency band. Thus, converging evidence suggests a distinct functional implication of the oscillatory activity in the delta-theta frequency band in visual processing.

We aimed here at understanding how these various oscillatory activities on different time scales are interrelated to each other, and what might be their functional role. A potential mechanism for cross-frequency interactions could be implemented by phase-amplitude coupling, (PAC) i.e., modulation of the amplitude of faster oscillations by the phase of slower oscillations (Jensen and Colgin, 2007; Canolty and Knight, 2010). This type of coupling has recently been found ubiquitously in various brain regions across a number of species (Bragin et al., 1995; Lakatos et al., 2005; Canolty et al., 2006; He et al., 2010), including visual cortices of macaques (Lakatos et al., 2005) and humans (Osipova et al., 2008; Händel and Haarmeier, 2009). Though the relevance of such cross-frequency coupling in brain functioning is yet to be elucidated, one intriguing hypothesis is that it provides a mechanism for the coordination of fast, spike-based computation with slower, external sensory and motor events (Canolty and Knight, 2010; Giraud and Poeppel, 2012). Thus, such a mechanism may be at work in free viewing which continuously requires such coordination of sensory (visual) inputs with motor action, as do occur during EMs.

With the aim of elucidating the interaction between the EM-related LFP activities on different time scales, we examined the changes in the spectral power and phase of LFP oscillations in monkey V1, in relation to self-initiated saccades during free viewing of natural-scene images. We found that the delta-theta band oscillations of the LFP are phase-locked to the timing of voluntary EMs, and that the degree of this phase-locking is strongly correlated with the degree of LFP power modulation in higher frequency bands, as expected from the hypothesis of PAC.

## Materials and methods

### Experiments

All experiments followed institutional and NIH guidelines for the care and use of laboratory animals. The experimental procedures are documented in detail in previous papers (Maldonado et al., 2008; Ito et al., 2011). Here, we therefore summarize key aspects of the experiments. Four capuchin monkeys (referred to as D, S, M, and G) participated in the experiments, where they were presented with a series of natural-scene images. Each image was presented for 3–7 s (depending on the monkeys) interleaved by a blank screen with a fixation spot. A computer monitor (frame rate: 60 Hz) located 57 cm in front of the animals, subtending 40 × 30° of visual angle, was used for the presentation of natural-scenes and visual control stimuli. The monkeys were allowed to make self-initiated EMs during the natural-scene image presentation, and rewarded with a drop of juice if they maintained their gaze within the edges of the monitor during the presentation. Upon the presentation of the blank screen with a fixation spot, they were required to keep their gaze on the fixation spot for 1 s to be rewarded. This was relevant only for maintaining the attention of the monkeys during the experiment, and hence the data recorded during this period of the experiment was not used in the current analysis. The total number of natural image presentations for each monkey is as follows: 427 presentations for monkey D, 776 presentations for monkey S, 793 presentations for monkey M, and 145 presentations for monkey G.

### Data collection

#### Eye-movement recordings

For the recording of the EMs, we implanted a scleral search coil in one eye of each monkey (Judge et al., 1980). Vertical and horizontal eye positions were monitored with a search coil driver (DNI Instruments, Resolution: 1.2 min of arc), and then stored in a hard disk at 2 kHz sampling rate. The onsets of saccades and fixations were extracted based on the following definitions. Saccades were defined as EMs with an angular velocity higher than 100°/s and lasting for at least 5 ms. In addition, saccades were required to exhibit a minimum acceleration of 170°/s^2^. The onset and the offset of saccades were defined as the moments when the velocity threshold was crossed from below and above, respectively. Saccade duration was defined as the interval between the onset and the offset of a saccade. Post-saccade periods were classified as fixations when they lasted at least 100 ms with the eye position maintained within 1° off of the gaze location reached at the end of the preceding saccade. Sustained movements with angular velocities ranging from 70 to 150°/s and durations of at least 100 ms were classified as drifts, the neuronal recordings during which we did not analyze in the present study. The total numbers of detected saccades for the four monkeys were 2291, 2365, 11,467, and 1448, for monkeys D, S, M, and G, respectively.

#### LFP recordings

LFP signals in the primary visual cortex were recorded with an array of eight individually adjustable custom fabricated nichrome tetrodes (1–2 MΩ impedance). The tetrodes were positioned in a circular array, with a center to center distance of ~400 μm. LFP signals were selected from the first electrode of each tetrode. As reference we utilized a metal screw anchored in the medial line of the occipital area of the skull. The signals were amplified (10 K), band-pass filtered (1–300 Hz) and then stored in an electronic device at 2 kHz sampling rate. A notch filter was applied off-line to the LFP signals in order to remove the 50 Hz noise of the power line. Thus, in one recording session we sampled neuronal signals from 8 recording sites, and we performed about 20–100 recording sessions per monkey. The total number of recording sites for each monkey is as follows: 274 sites for monkey D, 388 sites for monkey S, 822 sites for monkey M, and 120 sites for monkey G.

### Data analysis

#### Inter-saccade interval and saccade frequency

We denote the series of saccade onset times during one trial, i.e., a 3–7 s image presentation, as τ_*i*_ (1 < *i* < *N*), where *N* is the total number of the saccades performed during the trial. Inter-saccade intervals (ISIs) are defined as the intervals between successive saccade onsets, i.e., τ_*i* + 1_ − τ_*i*_ (1 < *i* < *N* − 1). For each of the monkeys, we collected ISIs from the whole recording sessions and calculated their histogram and median. The inverse of the median ISI was considered as saccade frequency, i.e., an estimate of the repetition rate of saccades.

#### Extraction of power and phase of LFP oscillations

For the estimation of the instantaneous power and phase of the LFP signals we used a wavelet transform (WT) with Morlet wavelets, defined at frequency *f* and time *t* by $\sqrt{f}\cdot \exp\left[i2\pi f\left(u-t\right)\right]\text{exp}\left[-{\left(u-t\right)}^{2}/(2\sigma^{2})\right]$ (Le Van Quyen et al., 2001). The parameter σ was set to 5/(6*f*), so that a wavelet contains about 5 cycles of oscillations. The center frequency *f* of the wavelet was varied within a range between 1 and 256 Hz, starting from 1 Hz and multiplied by 1.2 until it exceeded 256 Hz. This results in a uniform sampling of frequencies on a log scale. From the obtained WT at time *t* and frequency *f*, denoted as *S*(*t*, *f*), we extracted the instantaneous power *A*(*t*, *f*) and the phase φ(*t*, *f*) as the squared norm (*A*(*t*, *f*) = [Re *S*(*t*, *f*)]^2^ + [Im *S*(*t*, *f*)]^2^) and the argument (φ(*t*, *f*) = atan2[Im *S*(*t*, *f*), Re *S*(*t*, *f*)]) of the WT, respectively.

#### Saccade-onset triggered average of instantaneous LFP power

To study the modulations of the instantaneous LFP power in relation to saccadic EMs, we computed the time-resolved average of the instantaneous power triggered by saccade onsets. To do so, we first normalized the LFP power time series to the z-score using the mean and the standard deviation of the LFP power across all trials. Then these segments of the instantaneous LFP power time series, denoted as *A*_*i*_ (τ, *f*) = *A*(τ_*i*_ + τ, *f*), where τ_*i*_ is the *i*-th saccade onset time and τ taken as −100 to 300 ms around saccade onset, were averaged to yield the averaged power $\bar{A}(\tau ,f)=(1/N)\sum_{i=\;1}^{N}A_{i}(\tau ,f)$. The parameters τ and *f* are the variables for the time in relation to saccade onset and the frequency of the WT, respectively, constituting the axes of the time-frequency plots shown in Figure 3.

#### Saccade-onset triggered inter-saccade phase consistency of instantaneous phase

To study the locking of LFP oscillations to the onset of saccadic EMs, we calculated the inter-saccade phase consistency (ISPC) of the oscillation phase, which quantifies how coherent the phases are across saccades, at each time step (in relation to saccade onset) and each frequency, defined as ISPC(τ, *f*) = (1/*N*)|∑^*N*^_*j* = 1_ exp [*i*φ_*j*_ (τ, *f*)]|, with φ_*i*_(τ, *f*) = φ(τ_*i*_ + τ, *f*) (−100 < τ < 300 [ms]). As for the LFP power, the parameters τ and *f* are the time in relation to saccade onset and the frequency of the WT, respectively, and they constitute the axes of the time-frequency plots shown in Figure 4.

#### Definition of frequency components

In the further analyses we focused on four distinct frequency components which were defined separately for each of the monkeys based on their time-frequency profiles of the LFP power and the IPSC. The center frequency of the first component was defined to be the saccade frequency of each monkey. The center frequencies of the second and the third components are defined as the frequencies (rounded to integer values) at which two peaks of the LFP power were located at 100 ms after saccade onset, and thus vary across monkeys. The center frequency of the last component was taken to be 180 Hz for all monkeys. The frequencies were defined as the center frequencies obtained by the WT of the LFP recordings at these frequencies with the same mother wavelet and the same way to define the value of the parameter σ as described in “Extraction of Power and Phase of LFP Oscillations.” We termed these four components as delta-theta, alpha-beta, low-gamma, and high-gamma components, respectively. Their center frequencies are summarized in Table 1. The band widths of these components are proportional to their center frequencies *f*_*c*_ and can be derived approximately as [*f*_*c*_ − 1/(π σ), *f*_*c*_ + 1/(π σ)].

**Table 1:** **Center frequencies of the frequency components identified by the time-frequency analysis of the LFP power modulation and the inter-saccade phase consistency for each of the monkeys**.

**Frequency component**	**Center frequency**			
	**Monkey D (Hz)**	**Monkey S (Hz)**	**Monkey M (Hz)**	**Monkey G (Hz)**
Delta-theta	3.40	2.49	3.98	4.38
Alpha-beta	10	13	10	10
Low-gamma	20	32	32	40
High-gamma	180	180	180	180

#### Correlation between the ISPC and the average LFP power

We examined whether the variability in the delta-theta band ISPC values across recording sites is related to that of the LFP power values of the other frequency components by cross-correlating these measures. For each recording site, we sampled the mean ISPC values and the mean LFP power values in intervals 0–150 ms (for the delta-theta component), 50–150 ms (for the alpha-beta and the low-gamma components), and 100–200 ms (for the high-gamma component) from saccade onset. These time ranges were determined so that they are centered at the peak timing of the respective measures and include the whole episode of their EM-related modulations. Pearson's correlation coefficients were calculated between the delta-theta band ISPC and the LFP power of all frequency components. Significance of the obtained correlation coefficients deviating from zero was tested by computing the two-sided *p*-values of the coefficients based on the t-distribution with the corresponding degree of freedom (i.e., sample size −2).

## Results

### Eye movements

**Figure 1:**
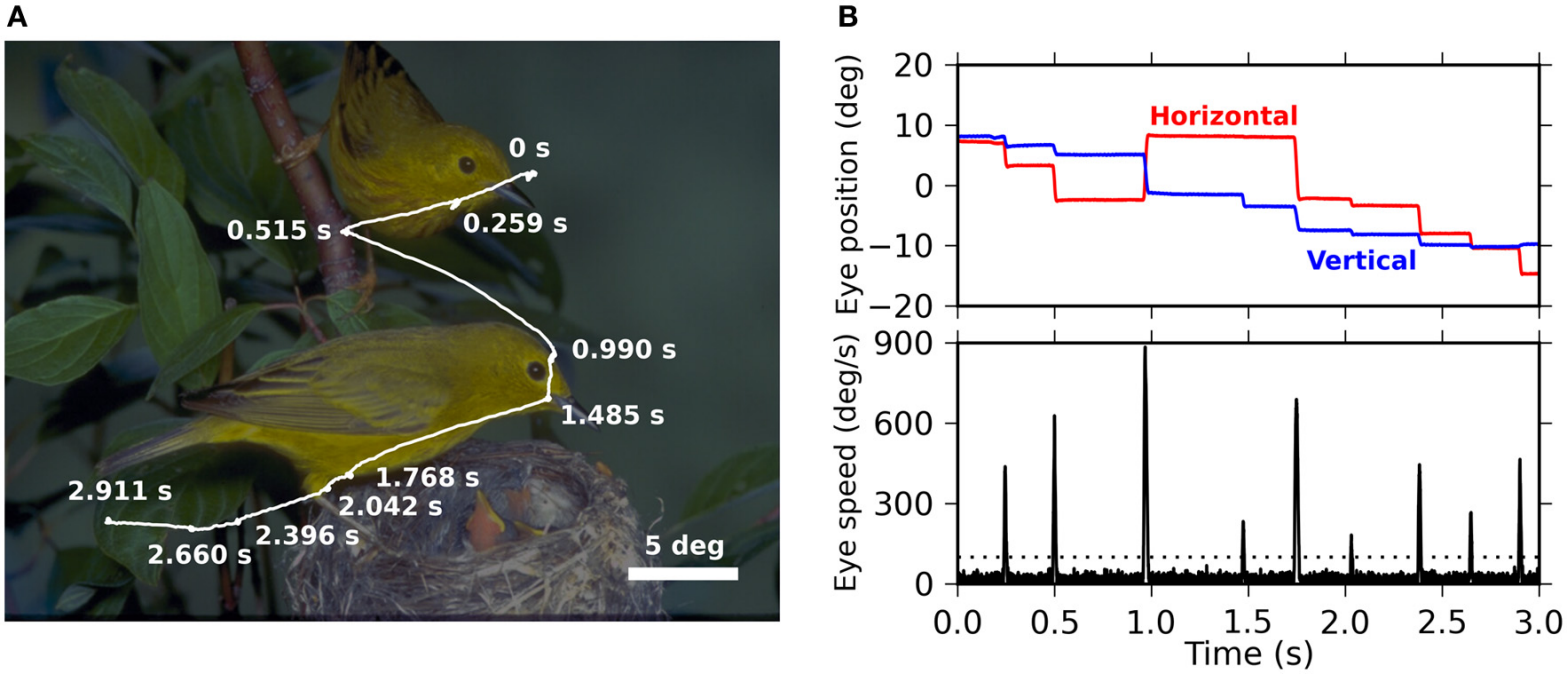
**(A)** Representative trace of eye movements performed by monkey D during free viewing of a natural-scene image (3 s presentation). The trace (white) is superimposed on the presented image, which is darkened for the purpose of a better visibility of the trace. Fixation points, seen as “knots” along the trace, are marked by their onset times measured from the image onset. **(B)** The top panel shows the time series of the horizontal (red) and the vertical (blue) eye positions during the eye movements shown in **(A)**. The origin, i.e., 0° for the horizontal and the vertical directions, is the center of the image. The bottom panel shows the velocity of the eye movements as derived from the eye positions shown in the top panel. The dotted line indicates the threshold for the detection of saccades (see “Materials and Methods” for the detection criteria).

As reported in previous papers (Maldonado et al., 2008; Ito et al., 2011; Berger et al., 2012), the monkeys voluntarily made exploratory EMs when they were presented with natural-scene images. Figure 1A shows a typical trace of the EMs performed by the monkeys. The time courses of the eye positions in horizontal and vertical directions are shown in Figure 1B, together with the eye velocities derived from them by calculation of temporal derivative. Saccades appear as “spikes” of high velocities (lower panel).

**Figure 2:**
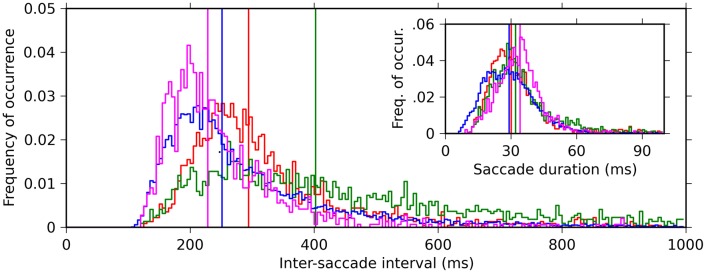
**Histograms of the inter-saccade intervals (ISI) (main plot, 5 ms bin) and saccade durations (inset, 1 ms bin) for the four monkeys (color code: red, green, blue, and magenta for monkeys D, S, M, and G, respectively).** Vertical lines indicate the medians of the respective histograms.

We recorded EMs from four monkeys while they performed free viewing of natural-scene images. The onsets and offsets of the saccades were detected based on the velocities and accelerations of the EMs (see “Materials and Methods” for the details). Note that in our definition the onset of a saccade is identical to the offset of the previous fixation, and the offset of a saccade is identical to the onset of the following fixation. To characterize the dynamics of the EMs, we computed the distribution of the intervals between the onsets of successive saccades (ISIs). In all four monkeys, the ISIs exhibited a unimodal distribution with a long tail toward longer intervals (Figure 2, main plot). The median of the ISIs of the individual monkeys varied in a range between 220 and 400 ms. We also computed the distribution of saccade durations, defined as the time interval between onset and offset of a saccade. The saccade duration distributions were more consistent across the monkeys than the ISI distributions: the median saccade durations were about 30 ms for all four monkeys (Figure 2, inset). This means that the differences of the median ISIs are mostly due to differences in the durations of fixations.

As a measure of the time scale of the saccade repetitions performed by the monkeys, we defined *saccade frequency* as the inverse of the median ISI. The saccade frequencies of the individual monkeys all lay within the delta-theta frequency band; specifically, 2.49, 3.40, 3.98, and 4.38 Hz for monkey S, D, M, and G, respectively.

### Saccade-related changes in the oscillatory power of the LFP

LFP signals from the primary visual cortex were recorded concurrently with the EMs. To assess the modulation of the LFP activity related to the EMs, we first analyzed the temporal changes in the LFP power in relation to the onset of saccades. The LFP power was estimated using the Morlet WT, normalized (subtraction of the mean and division by the standard deviation) separately for each frequency in the range of 1–256 Hz, and then averaged across individual saccades with the saccade onset as trigger (see “Materials and Methods” for analysis details).

**Figure 3:**
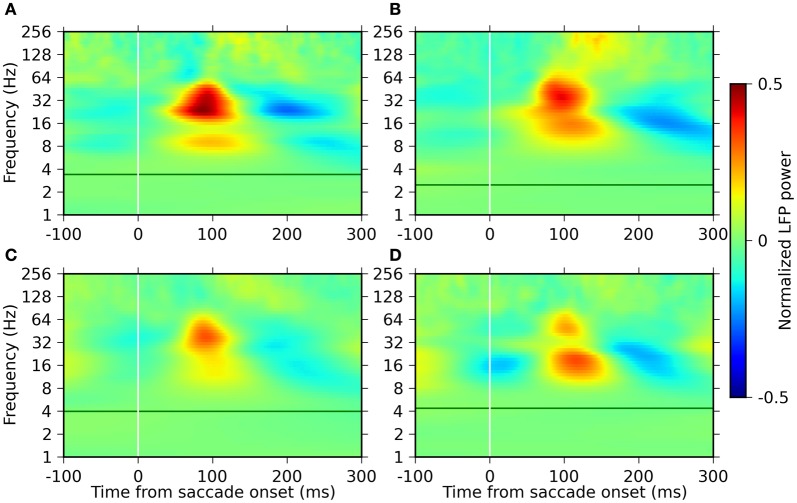
**Time-frequency plots of the LFP power modulation around saccade onset for the four monkeys.** Panels **(A–D)** represent the results of monkeys D, S, M, and G, respectively. The power is normalized (mean-subtraction and division by the standard deviation) separately for each frequency and plotted in pseudo-color code indicated by the color bar on the right. The green horizontal lines indicate the saccade frequency of the individual monkeys.

The resulting time-frequency profiles of the LFP power modulations (Figure 3) were consistent across four monkeys. In all the monkeys, noticeable changes in the power were restricted to frequencies above ~8 Hz. This result is reasonable given the following argument. An estimation of the instantaneous power of an oscillation requires an observation lasting for roughly one cycle of an oscillation. Accordingly, that the waxing and waning of the oscillatory power becomes visible within one fixation period (200–400 ms on average) requires more than two cycles contained within this time period. This requirement can only be met for oscillations faster than ~8 Hz.

The most prominent modulation in power is observed in the frequency range between 8 and 64 Hz. Two separate components are present within this range, with frequency bands and their peak frequency varying across the monkeys, but on average covering one band of 8–32 Hz and the other of 16–64 Hz. Both of these components exhibit an increase in power starting at around 50 ms after saccade onset, reaching the peak at around 100 ms, and are then followed by a period of reduced power.

Another component of power modulation is observed in a frequency band above ~100 Hz. The power of this component starts to increase at around 100 ms, which is considerably later than the components in the lower frequency ranges (8–64 Hz). The degree of the power modulation of this component was much smaller than those of the lower frequency components.

### Saccade-related phase-locking of LFP oscillations

We analyzed the relation of the phase of the oscillation to the timing of EMs. We estimated the phase of the LFP oscillations using the Morlet WT and computed the phase consistency across saccades (ISPC; see “Materials and Methods” for analysis details). ISPC values were computed in a time-resolved manner aligned to saccade onset and separately for each frequency in the same ranges as used for the power modulation analysis.

**Figure 4:**
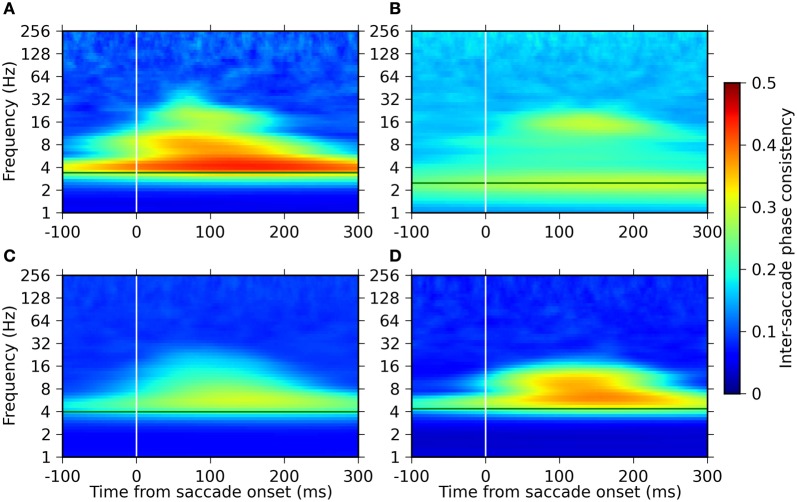
**Time-frequency plots of the inter-saccade phase consistency around saccade onset for the four monkeys.** Panels **(A–D)** represent the results of monkeys D, S, M, and G, respectively. The green horizontal lines indicate the saccade frequencies of the individual monkeys. The strength of the ISPC is indicated in a pseudo-color code (color bar on the right).

The time-frequency profile of the ISPC was, similarly to the power modulation, consistent across monkeys (Figure 4). All monkeys exhibited high phase consistencies at their respective saccade frequencies. The highest ISPC value in this frequency band was observed at 100–200 ms after saccade onset. In addition, we found another ISPC component in a frequency range between 8 and 32 Hz, which roughly corresponds to the lower one of the two power modulation components in the range of 8–64 Hz. No significant phase consistency was observed in the frequencies above 64 Hz.

Based on the above observations of the time-frequency profiles of the power and the IPSC, we defined four distinct frequency components of EM-related LFP activity (see the “Materials and Methods” for the detailed definition procedure). The lowest component is defined to be centered at the saccade frequency of each monkey. This component is characterized by the strong phase consistency observed in all animals. The other three components are centered at the frequencies where the power modulation is maximal. The center frequencies of the second and the third lowest components are defined as the frequencies of the maximum LFP power at 100 ms after saccade onset, and thus vary across monkeys. The center frequency of the highest component is taken as 180 Hz for all monkeys. We termed these components as delta-theta, alpha-beta, low-gamma, and high-gamma components from the lowest to the highest frequency. The center frequencies of these components are summarized for each of the monkeys in Table 1.

### Correlation between the power and ISPC values across frequencies

**Figure 5:**
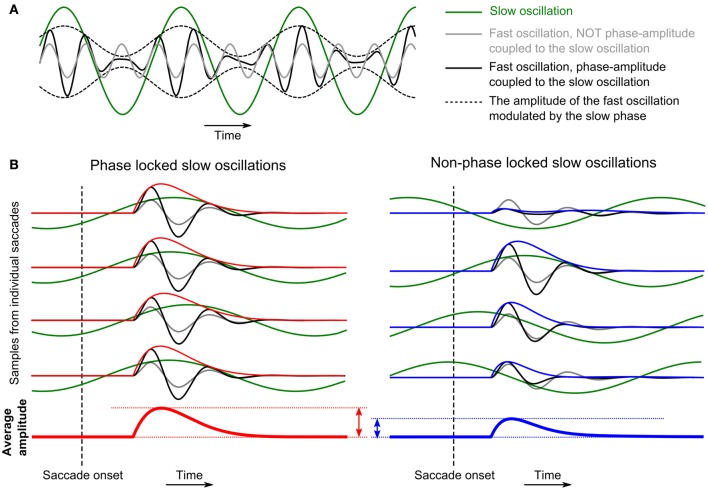
**(A)** Schematic representation of the effect of phase-amplitude coupling (PAC) between slow and fast oscillations. In this example, the amplitude of the fast oscillation is enhanced (or suppressed) at the peak (or the trough) of the slow oscillation. **(B)** Schematic representing how PAC causes correlation between phase-locking of the delta-theta band oscillations to the timing of EMs and the average power in the alpha-beta or the low-gamma band evoked by the EMs.

The power and ISPC values computed from recordings in different sessions, i.e., at different electrode positions, exhibited variability around the average values that are shown in Figures 3 and 4, and it sometimes became very large even across simultaneous recordings in an identical recording session. We examined whether this variability is correlated across different frequency components; in other words, whether there are interactions between the different frequency components. Recent studies on cross-frequency interactions have reported modulations of the amplitude of fast oscillations in relation to the phase of slow oscillations (PAC or nested oscillations; Bragin et al., 1995; Lakatos et al., 2005; Canolty et al., 2006; He et al., 2010; see sketch in Figure 5A). If we assume that also here such an interaction exists, in particular between the phase of the delta-theta band oscillations and the amplitudes of the other frequency components, then, it is expected that the amplitude of the fast oscillations averaged across saccades would be positively correlated with the ISPC of the slow oscillations. In the case where the ISPC is high (Figure 5B left), the positive modulation of the fast amplitude by the slow phase would occur at a consistent timing in relation to the timing of EM, while, in the case where the ISPC is low (Figure 5B right), such amplitude enhancement would occur at arbitrary timings and hence the average amplitude becomes small compared to the other case. As a consequence, by examining the correlations between the delta-theta ISPC values and the powers in the other frequency components, we can infer which frequency pairs are phase-amplitude-coupled.

**Figure 6:**
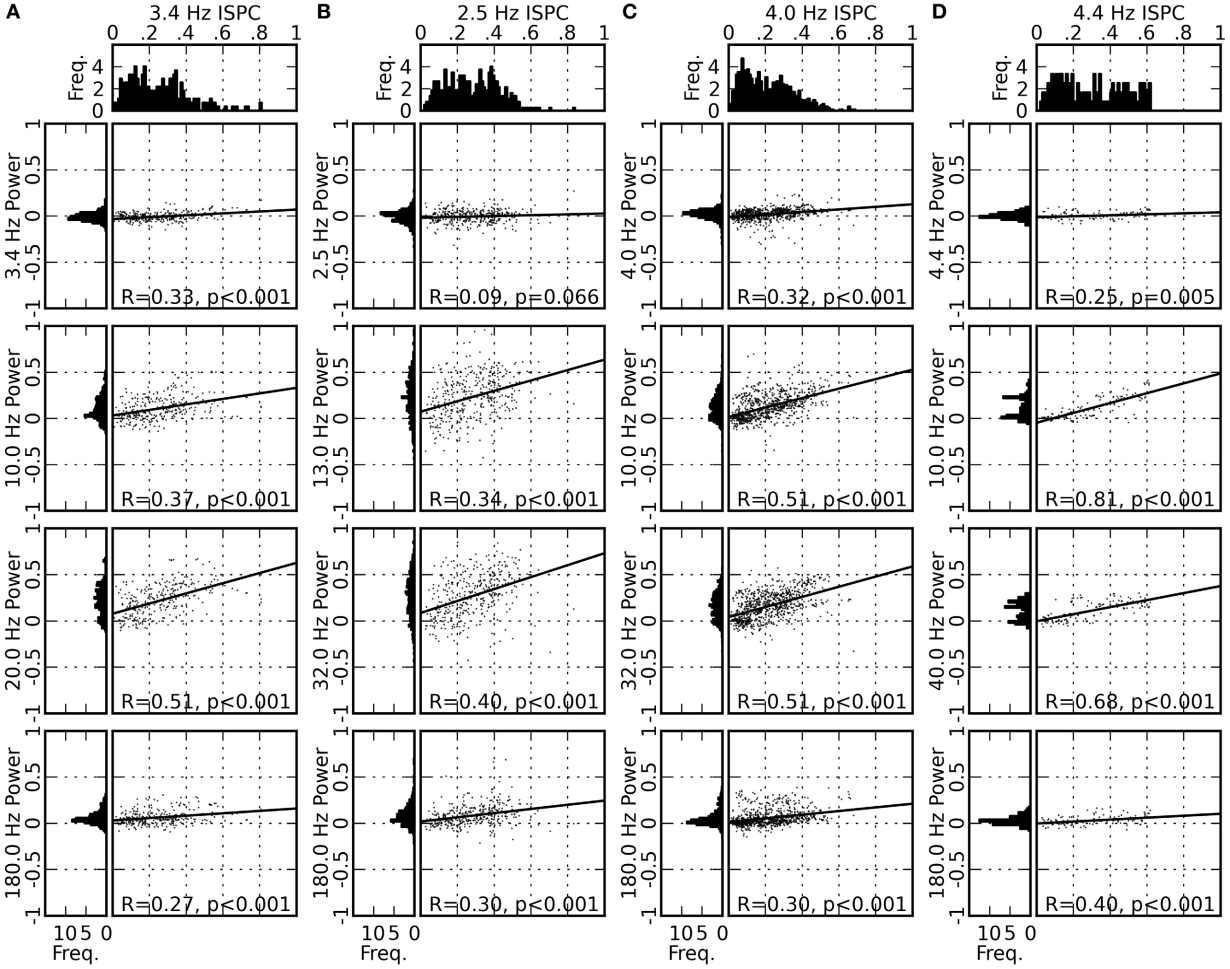
**Distribution of the ISPC values for the delta-theta band (top panels), distribution of the normalized LFP power for four frequency bands (left columns; same normalization as in Figure 3), and scattergrams of the ISPC and the LFP power values for all pairings of the four frequency bands (right columns).** The horizontal and vertical position of a single dot in a scattergram represent the delta-theta ISPC value and the LFP power in the corresponding frequency band, respectively, obtained from the data from one recording site in one of the experimental sessions. Results from all recording sites in all experimental sessions are plotted altogether. The solid line represents the linear regression of the data points, with its correlation coefficient (*R*-value) and the *p*-value marked at the bottom (see “Materials and Methods” for methodological details). Panels **(A–D)** represent the results for monkeys D, S, M, and G, respectively.

To test the outlined hypothesis, we analyzed the variability of the spectral power and the ISPC values across different recording sites. We first computed the mean ISPC values of the delta-theta band component between 0 and 200 ms after saccade onset separately for each of the single recording sites. The distribution of the obtained mean ISPC values (Figures 6A–D, top panels) is broad, indicating a large variability of the delta-theta band ISPC across different recording sites. We then computed the same kind of distribution for the LFP power for each frequency component (Figures 6A–D, left columns). The distributions for the alpha-beta band and the low-gamma band components are found to be wide, including large positive values and a mean larger than zero, while the distributions for the delta-theta and the high-gamma band components are centered around zero and are narrowly peaked.

We further examined whether the variability in the delta-theta band ISPC is correlated to the variability of the power in other frequency bands. Figures 6A–D (right columns) shows scatter diagrams of the power and the ISPC values for all pairs of frequency components. Positive correlations are found for all comparisons, with slopes significantly different from zero for most of the cases. Strongest correlations, in terms of both the slope of the linear regression and *R*-value, are found between the delta-theta ISPC and the alpha-beta or the low-gamma band power, suggesting cross-frequency coupling between the phase of the delta-theta band and the saccade-related power modulation in the alpha-beta or the low-gamma band. In the Discussion section we will provide possible interpretations of these results and discuss a plausible mechanism underlying these observations.

### Timing relationship of LFP signals to saccade- and fixation-onsets

**Figure 7:**
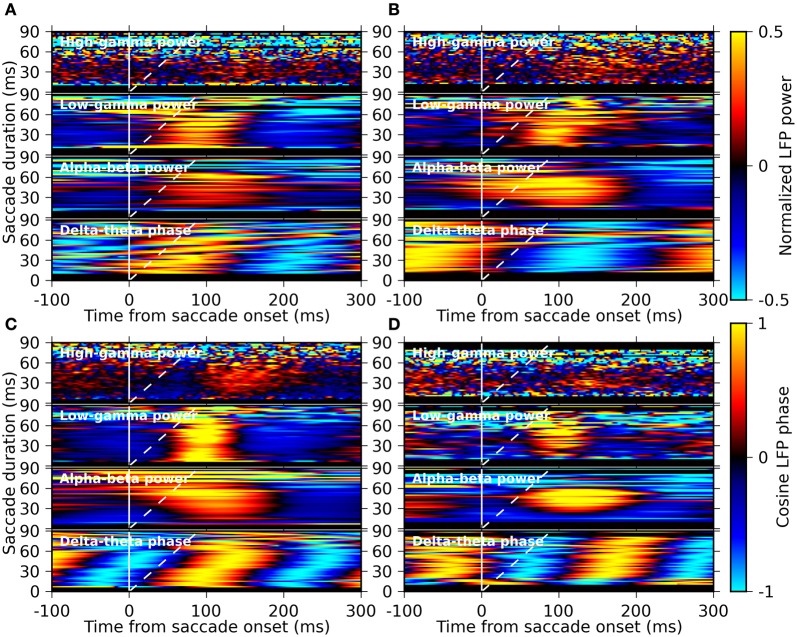
**Saccade-duration resolved plots of the average LFP phase and average LFP power in different frequency bands triggered on saccade onset.** Panels **(A–D)** represent the results for the monkeys D, S, M, and G, respectively. In each panel, the average phase of the delta-theta band component and the average power of the alpha-beta, the low-gamma, and the high-gamma frequency components are shown from bottom to top, respectively. The averages were computed separately for sets of saccades with different saccade durations (bin width: 2 ms), constituting the y-axis of each plot in pseudo-color (color bars on the right). The solid and dashed white lines indicate the timing of saccade-onset and fixation-onset, respectively, at each vertical position within each plot.

Ito et al. (2011) have recently shown that the EM-related LFP oscillations in the alpha-beta band are locked to saccade onset rather than to fixation onset. Here we examine this timing relationship by computing averages of the LFP power and the LFP phase resolved by saccade duration. Therefore, we computed the saccade-onset triggered average of the LFP phase for the delta-theta band component and of the LFP power for the other frequency components separately for different saccade durations (in steps of 2 ms bins). The results are shown as a function of time and saccade duration in Figure 7. We found that the delta-theta phase is largely locked to fixation onset rather than to saccade onset, as indicated by the oblique stripes of iso-phase domains, while the alpha-beta power and the low-gamma power are locked to saccade onset, which is most clearly seen for monkeys M and G (Figures 7C,D). This suggests that the delta-theta band LFP oscillations are engaged in different aspects of visual processing than those related to the alpha-beta and the low-gamma band oscillation.

## Discussion

We identified four frequency components of EM-related LFP activity based on the time-frequency characteristics of the LFP modulations in the primary visual cortex of monkeys performing voluntary visual exploration of natural-scene images. The center frequencies of the four components were found in the delta-theta band (2–4 Hz), the alpha-beta band (10–13 Hz), the low-gamma band (20–40 Hz), and the high-gamma band (>100 Hz). The strongest changes in the LFP power in response to EMs were observed in the alpha-beta and the low-gamma band components, while the phase-locking to the timing of EMs was strongest in the delta-theta band component. We found positive correlations between the degree of phase-locking in the delta-theta band and the magnitude of the power increase after EMs in the other frequency bands. This correlation was strongest for the alpha-beta and the low-gamma band power.

The strongest phase-locking within the delta-theta band was observed at slightly different frequencies for different monkeys, but these frequencies systematically matched the individual saccade frequencies, i.e., the inverse of the median ISI. This result is consistent with a previous finding on the phase-locking of the delta-theta band LFP oscillations in V1 and V4 to the onset of micro-saccades during prolonged fixation (Bosman et al., 2009). This consistency offers a strong support for the view that regular saccades and micro-saccades constitute a functional continuum of ocular movements that influence the visual cortex (Otero-Millan et al., 2008; Hafed et al., 2009). Our results about the delta-theta band phase-locking are also consistent with previous findings on the delta-theta band LFP phase during passive viewing of natural movies (Belitski et al., 2008; Mazzoni et al., 2011). These studies found that the LFP phase in this frequency band contains information about the slow fluctuations of the luminance of a presented movie. The authors argued “Because movies contain most power at low frequency, it is conceivable that some of the features for which LFPs are selective are characterized by slow fluctuations and thus are reflected in LFPs at low frequency” (Belitski et al., 2008). We assert that, in natural vision, the temporal changes in the afferent input to the visual system on this time scale are caused by voluntary saccadic EMs, even if there are no movements in the external visual world. Thus, the phase-locking of the delta-theta band LFP oscillation in the present study is considered as an extension of the previous finding in passive movie viewing to the condition of active visual exploration.

The degree of the observed phase-locking in the delta-theta band was not homogeneous across different recording sites, but it was highly variable. We could not find any systematic relation between the strength of the phase-locking and (a rough estimate of) the recording depth within the cortical layers. This variability may be explained by the differences in the response properties of the local neuronal populations across V1 and/or the statistical properties of the natural image stimuli we used. An elucidation of this issue would require further experimentation.

Modulation of oscillatory power in relation to EMs was observed in the alpha-beta, the low-gamma, and the-high gamma frequency bands. The increase in power in the alpha-beta band is consistent with our previous results (Ito et al., 2011). The power modulations in the low- and the high-gamma bands, and their temporal relation to that in the alpha-beta band are novel findings of the present study. The low-gamma component shares a common time course of power changes with the alpha-beta component, while the high-gamma component shows a clearly different time course in the changes than the others. The degree of the power changes is larger in the alpha-beta and the low-gamma bands than in the high-gamma band. The phases of the high- and the low-gamma components are not locked to the onset of EMs, which is a signature of induced oscillations, while the power increase in the alpha-beta component is associated with phase-locking, which is a signature of evoked oscillations. All these differences in response properties across different frequency components strongly suggest that the neuronal activity in V1 during active vision is composed of multiple oscillatory components which have different mechanisms of generation.

The peak values of the power in response to EMs showed a large variability across recording sites, as was also found for the ISPC of the delta-theta component. We found that the variability in the ISPC and the power were not independent, but show positive correlation between the delta-theta band ISPC and the power of the other frequency components derived from identical electrodes. This correlation was particularly strong for the alpha-beta and the low-gamma power. A parsimonious interpretation of this result is that the delta-theta, the alpha-beta, and the low-gamma components are just the reflections of an identical physiological process that possesses power in a wide frequency range (for example, evoked oscillatory activity with a non-sinusoidal waveform). However, the results of our saccade-duration resolved analysis argue against this view. We found that the delta-theta phase is locked more to fixation onset than to saccade onset, while the power of the alpha-beta and the low-gamma is locked more to saccade onset. This strongly suggests that there are at least two separate underlying neuronal processes that are related to the onset of fixations and that of saccades, and that the former is responsible for the generation of the delta-theta component and the latter for the other (the alpha-beta and the low-gamma) components. Furthermore, recent studies have shown that, while the power of the high-gamma broad band activity reflects the amount of the spiking activity of local neuronal pools, the power in lower frequencies is more related to network oscillations (Ray et al., 2008; Ray and Maunsell, 2011). We found that the phase-locking of the delta-theta component is more strongly correlated to the power of the low-gamma component than to the power of the high-gamma component. This suggests that the LFP activity in the delta-theta band is not a mere reflection of the changes in the spiking activity of the local neuronal pool, but it would be related to network oscillations of neuronal excitability.

As illustrated in Figure 5, the observed correlation between the phase-locking in the delta-theta band and the evoked power in the alpha-beta and the low-gamma bands can be interpreted as cross-frequency interactions occurring via the mechanism of PAC (Jensen and Colgin, 2007; Canolty and Knight, 2010). Our observations of the ISPC-power correlation can be explained as a reflection of PAC between the slow LFP oscillation at the saccade frequency and the fast-evoked LFP oscillations in the alpha-beta or the low-gamma frequency band. Under the assumption of such PAC, a recording with high delta-theta ISPC would be associated with strong alpha-beta or low-gamma power, since the enhancement of the alpha-beta or low-gamma amplitude at a proper delta-theta phase occurs at a consistent timing in relation to the timing of EM, which results in a high-evoked power on average across EMs (Figure 5B left). On the other hand, if the delta-theta phase is not locked to the timing of EMs, such amplitude enhancement occurs at arbitrary timing and hence the average-evoked power becomes smaller compared to the case with strong phase-locking (Figure 5B right). A possible mechanism underlying such cross-frequency interaction is, as proposed by Mazzoni et al. (2008, 2010), a modulation of the baseline excitability of V1 neurons by unspecific slow cortical activity. In natural vision, such a slow activity could be a top-down, predictive signal entrained to the rhythmic EMs (Lakatos et al., 2008), or could originate from the LGN activity that is rhythmically modulated by a corollary signal derived from the motor commands to the eye muscles (Wurtz et al., 2011). Recent studies have reported the evidence that visual attention is temporally modulated at the theta band rhythm (Landau and Fries, 2012) and that such modulation is mediated by cross-frequency interaction between the theta and the gamma band LFP activities (Bosman et al., 2012). Since EMs are tightly related to visual attention (Corbetta et al., 1998), the EM-related cross-frequency interaction between the delta-theta and the higher frequency components identified in the present study could be a candidate mechanism for modulation of attention during natural vision with voluntary EMs.

Previous studies on natural viewing have shown that firing rates of V1 neurons during exposure to complex scenes are characteristically low (Gallant et al., 1998; Vinje and Gallant, 2000; Olshausen and Field, 2005; MacEvoy et al., 2008; Maldonado et al., 2008). For example, Maldonado et al. (2008) reported that the peak firing rate during visual fixations is on average ~15 Hz. Under such a condition, rate coding, i.e., information coding by spike counts during an certain period, would be unreliable, since given the low firing rates the number of spikes within one fixation would be at most 3–5 spikes and hence any additional spontaneous spiking during a fixation period could considerably alter the information content. This perspective is also supported by theoretical and experimental works that have proposed that information may not be encoded solely in firing rates but also in the precise and coordinated timing of action potentials, such as in the response latency (Gawne et al., 1996; Reich et al., 2001; VanRullen and Thorpe, 2002) or the spike timing in relation to background LFP oscillations (Montemurro et al., 2008; Nadasdy, 2009). We found in our previous studies that spike synchrony between V1 neurons exceeding chance synchrony predicted by the firing rates occurs and increases at around the onset of the rate change in response to visual fixation (Maldonado et al., 2008). In Ito et al. (2011) we additionally found that the first visually evoked spikes during fixations are locked to a specific phase of the LFP oscillations in the beta frequency band. Thus, the beta band LFP oscillations seem to provide a time-reference for spike synchrony among V1 neurons. Our present results suggest the enhancement of the alpha-beta power by phase-locking of the delta-theta oscillations to EMs. Taken together, these results point toward hierarchically organized brain activity in the temporal domain such that slower activities on the behavioral time scale influence the timing of single spikes via multiple levels of interaction between different time scales. Thus, experiments that employ voluntary, exploratory sensing behaviors by the animals provide the context for studying such temporal organization of neuronal activities and reveal the dynamic aspects of the sensory systems of the brain.

## Author contributions

Experiments and data acquisition was performed by Pedro Maldonado. Data analysis was made by Junji Ito and Sonja Grün. Manuscript was written by Junji Ito, Pedro Maldonado, and Sonja Grün.

## Grants

Financial support for this study was provided by funding from Iniciativa Cientifica Milenio P10-001-F and P09-015-F (Pedro Maldonado), the Helmholtz Alliance on Systems Biology (Sonja Grün, Junji Ito), and German-Japanese Joint Computational Neuroscience Program (BMBF grant 01GQ1114) (Sonja Grün, Junji Ito).

### Conflict of interest statement

The authors declare that the research was conducted in the absence of any commercial or financial relationships that could be construed as a potential conflict of interest.
